# Liver organoids: an in vitro 3D model for liver cancer study

**DOI:** 10.1186/s13578-022-00890-8

**Published:** 2022-09-09

**Authors:** Renshun Dong, Bixiang Zhang, Xuewu Zhang

**Affiliations:** 1 Tongji Hospital, Hepatic Surgery Center, Tongji Medical College, Huazhong University of Science and Technology, Wuhan, 430030 Hubei China; 2 Clinical Medical Research Center of Hepatic Surgery at Hubei Province, Wuhan, 430030 Hubei China; 3 Hubei Key Laboratory of Hepato-Pancreato-Biliary Diseases, Wuhan, 430030 Hubei China

**Keywords:** Liver organoid, Primary liver cancer, HBV infection

## Abstract

Primary liver cancer (PLC) is the second leading cause of cancer mortality worldwide, and its morbidity unceasingly increases these years. Hepatitis B virus (HBV) infection accounted for approximately 50% of hepatocellular carcinoma (HCC) cases globally in 2015. Due to the lack of an effective model to study HBV-associated liver carcinogenesis, research has made slow progress. Organoid, an in vitro 3D model which maintains self-organization, has recently emerged as a powerful tool to investigate human diseases. In this review, we first summarize the categories and development of liver organoids. Then, we mainly focus on the functions of culture medium components and applications of organoids for HBV infection and HBV-associated liver cancer studies. Finally, we provide insights into a potential patient-derived organoid model from those infected with HBV based on our study, as well as the limitations and future applications of organoids in liver cancer research.

## Introduction

Primary liver cancer (PLC) is the second leading cause of cancer mortality worldwide, and its morbidity has continuously increased in recent years [1]. PLC comprises hepatocellular carcinoma (HCC), intrahepatic cholangiocarcinoma (iCCA), and other tumors, such as hepatoblastoma and fibrolamellar carcinoma. HCC is the dominant type with nearly 800,000 new cases annually, accounting for 90% of PLC cases [2]. iCCA is the second most prevalent PLC, and its incidence continuously grows [2, 3]. Some studies have elucidated that adult hepatocytes and progenitor cells are the sources of HCCs and can transform into iCCAs, while adult cholangiocytes can only give rise to iCCAs [2]. However, more researches are needed to investigate the cell origin of HCC and iCCA [2, 4]. Both HCC and iCCA are heterogenous diseases among patients and even within cancer cells derived from the same individual. The importance of personalized medicine is increasingly prominent.

As in vitro models for cancer research, traditional two-dimensional (2D) cell line cultures and mouse models are frequently utilized for preclinical studies. Despite their unique contributions to the development of cancer science, they are also fraught with some challenges. For example, 2D cell lines lack the capability of mimicking the architectural features of tumors. Genetically engineered mouse models (GEMMs) and patient-derived xenografts (PDXs) are time-consuming and economically unaffordable for some researchers [1, 5]. Compared with traditional 2D models, three-dimensional (3D) systems are more adept at representing the situation in vivo (Table 1).

**Table 1:** Comparison for key features between 2D cell lines and 3D organoids

Comparison	2D cell lines	3D organoids
Morphology	Sheet-like flat monolayer	Self-organization, mimicking organ structure
Origin of cells	Cell line derived from a single cell	Related to tissues used for cell isolation
Heterogeneity	Unable to recapitulate critical features of the native tissues	Able to recapitulate structural, genetic, transcriptional and histological features of the native tissues
Resource costs	Low	High
Long-term expansion	Immortalized and easy expansion	Robust long-term expansion with maintenance of heterogeneity
Co-culture	Difficult; trans-well culture system allows co-culture of different cell types	Easy for co-culture of different cell types because of extracellular matrix

As a branch of 3D models in vitro, organoids play an increasingly pivotal role in cancer research. Although many definitions exist for organoid, this term is now defined as a “self-organizing structure that is generated from stem cells that mimics the in vivo architecture and multi-lineage differentiation of the original tissue” [6]. In the past decades, studies have reported the generation of organoids of several different organs, including the retina [7, 8], brain [9], kidney [10], lung [11], prostate [12], colon [13], pancreas [14, 15], liver [16], and some cancer organoids, such as brain tumors [17], pancreatic cancer [18], and PLC [19].

In this review, we summarize the categories and development of liver organoids, as well as liver organoid culture and applications in cancer research, mainly focusing on the functions of culture medium components and applications for hepatitis B virus (HBV) infection and HBV-associated liver cancer studies. In addition, we provide insights into a potential patient-derived organoid model from those infected with HBV based on our study, as well as the limitations and future applications of organoids in liver cancer research.

## Categories and development of liver organoids

The origin of organoids can be traced back to the research of silicious sponges by Wilson in 1907 [20]. He found sponges when kept in confinement under proper conditions degenerate, giving rise to small masses of undifferentiated tissue which in their turn are able to grow and differentiate into perfect sponges. It suggests the characteristic of self-organization exists in organisms, which is the key characteristic of organoids and the basis of organoid generation.

**Fig. 1:**
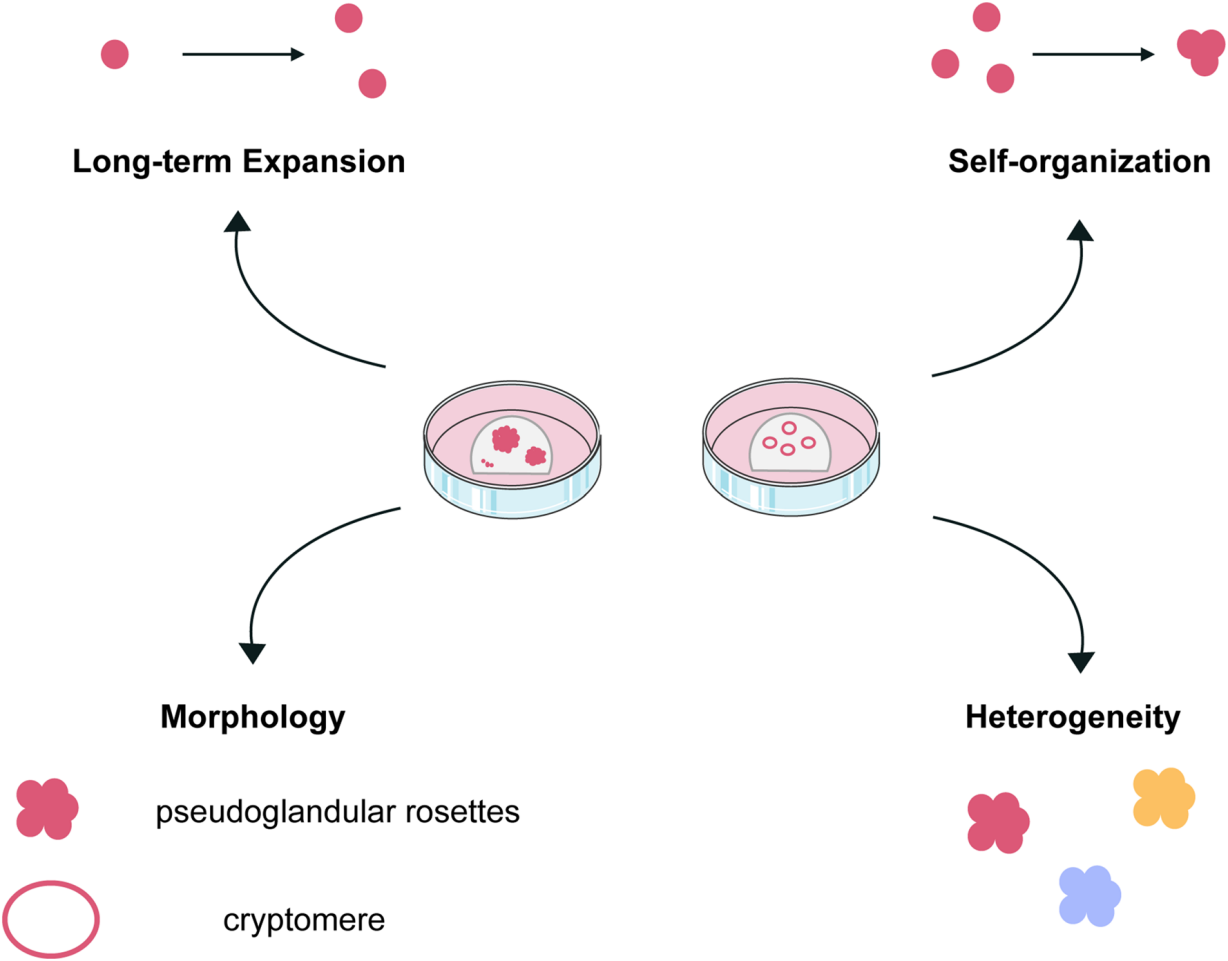
Features of liver organoids. As one kind of organoid models, liver organoids have the characteristic of long-term expansion and self-organization. In addition, liver organoids are of heterogeneity and with unique morphology features. Hep-Orgs are of pseudoglandular rosettes and Chol-Orgs are cryptomere

Organoids were once described as an in vitro 3D cellular cluster that can recapitulate the functionality of the origin tissue and are capable of self-renewal and self-organization [21, 22]. A landmark work in the field of organoids comes from the team of Hans Clevers in 2009. They have proven that there is a group of cells isolated from mouse intestinal tissue expressing the leucine-rich repeat-containing G-protein-coupled receptor 5 (Lgr5), and they are what we call Lgr5^+^ adult stem cells now. Based on these cells, organoids can be generated under special conditions with the characteristics of self-renewal and self-organization, that mimic the intestinal crypt-villus architecture and cell composition [23]. On the basis of this pioneering work, liver organoids get access rapidly from Lgr5^+^ mouse liver stem cells, although liver Lgr5^+^ cells can only be observed after chemical treatment induces liver damage [16] (Fig. 1).

**Fig. 2:**
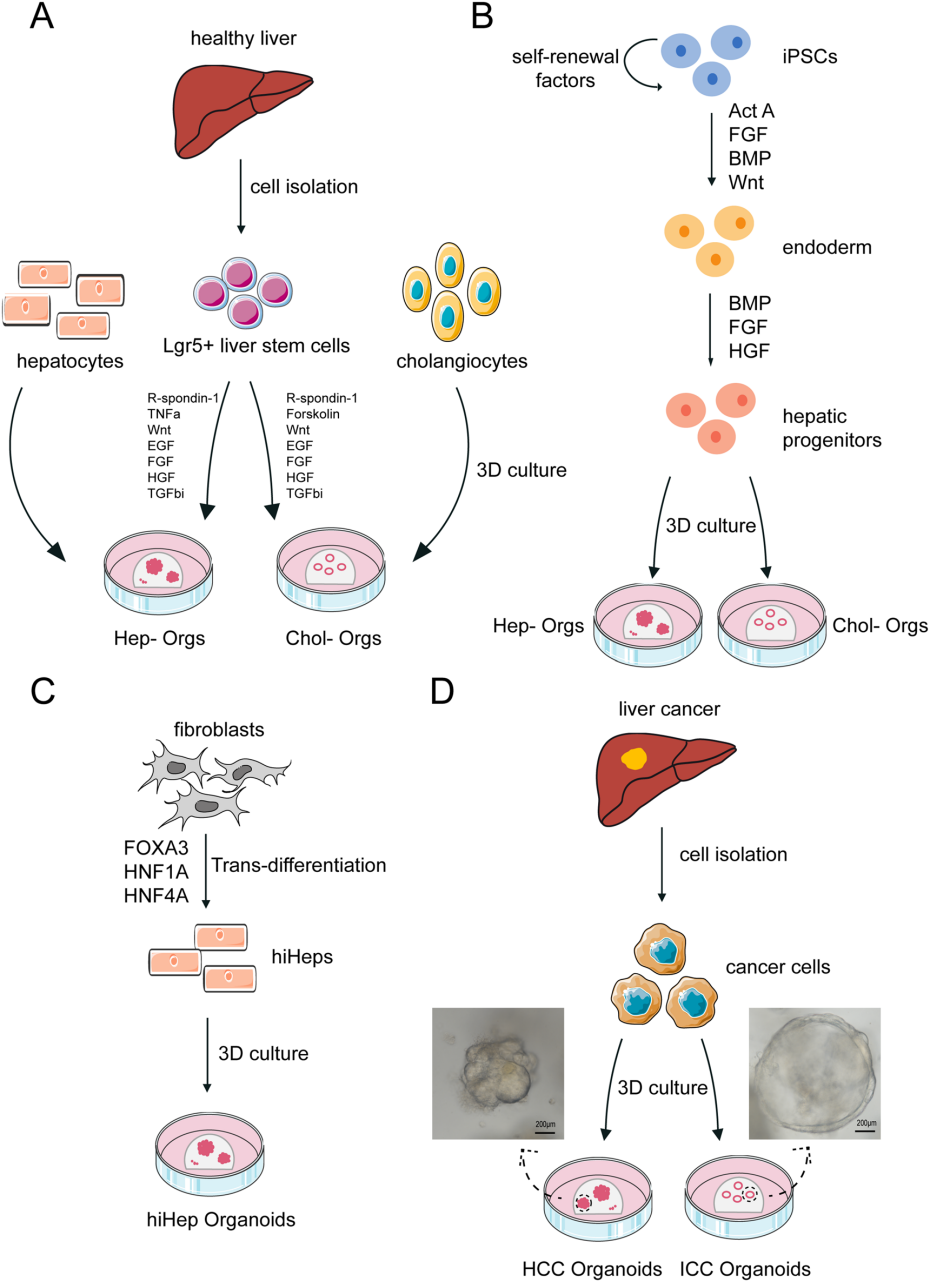
Liver organoids can be derived from various cells of origin. **A** Generation of liver organoids from healthy liver tissues. Liver organoids can be formed from hepatocytes, cholangiocytes and Lgr5^+^ liver stem cells. The isolated cells can be placed in Matrigel as extracellular matrix and seeded into culture vessels to generate organoids. Signaling pathways which are typically modulated to enable organoid formation are listed. **B** Generation of liver organoids from iPSCs. Liver organoids can be generated from iPSCs, usually by a three-stage differentiation process. Firstly, iPSCs can be derived to endoderm cells by exposure to Act A and Wnt. Then, these endoderm cells progress to a hepatic fate following induction of HGF and FGF signaling. These hepatic progenitors are hepatoblast-like cells and can form hepatocyte-like cells finally. **C** Generation of liver organoids from fibroblasts. Fibroblasts can be induced into hiHeps by exposure to FOXA3, HNF1A and HNF4A to activate trans-differentiation. Then, liver organoids can be generated from these hiHeps. **D** Generation of liver cancer organoids from liver cancer tissues. Similarly, liver cancer organoids can be formed from cancer cells isolated from cancer tissues. After seeding the isolated cells into culture vessels containing appropriate matrix, cancer organoids can be generated in the certain culture medium. *Act A* activin A, *BMP* bone morphogenetic protein, *EGF* epidermal growth factor, *FGF* fibroblast growth factor, *HGF* hepatocyte growth factor, *iPSCs* induced pluripotent stem cells, *TGFbi* transforming growth factor beta inhibitor, *TNFa* tumour necrosis factor-alpha

Liver organoids can be divided into two groups: healthy and cancer organoids (Fig. 2). The former are usually derived from stem cells and normal tissue cells, including hepatocytes, cholangiocytes, and even fibroblasts. Some studies have stated that primary hepatocytes and cholangiocytes [24] can be used to generate liver organoids in both mice and human [25, 26]. Some studies have demonstrated that transdifferentiation occurs, which means that fibroblasts can be converted into hepatocyte-like cells by activation of liver transcription factors [27–30]. Based on that technology, Sun et al. established liver organoids in ultra-low adherent plates [31]. As for stem cells, Lgr5^+^ liver stem cells and human induced pluripotent stem cells (iPSCs) have been used. Lgr5^+^ cells can be induced into bipotent progenitor organoids with the ability to differentiate into functional hepatocytes [16, 24]. Hepato-biliary organoids, a kind of complex organoids, can be generated by co-differentiation of hepatocytes and cholangiocytes from iPSCs [32, 33]. Based on the generation of organoids, carcinogenesis models could be established by chemical treatment and genetic engineering editing [34, 35].

When the establishment of cancer organoids achieves great success, the meaning of organoids expands. Unlike healthy organoids, cancer organoids mainly originate from cancer cells. It has been reported that primary cancer cells isolated from mouse models and patients can be induced into organoids under special conditions. Success has been achieved in the establishment of malignant organoid models from mouse primary liver tumors, after isolation and culture of PLC tissue [36].

**Fig. 3:**
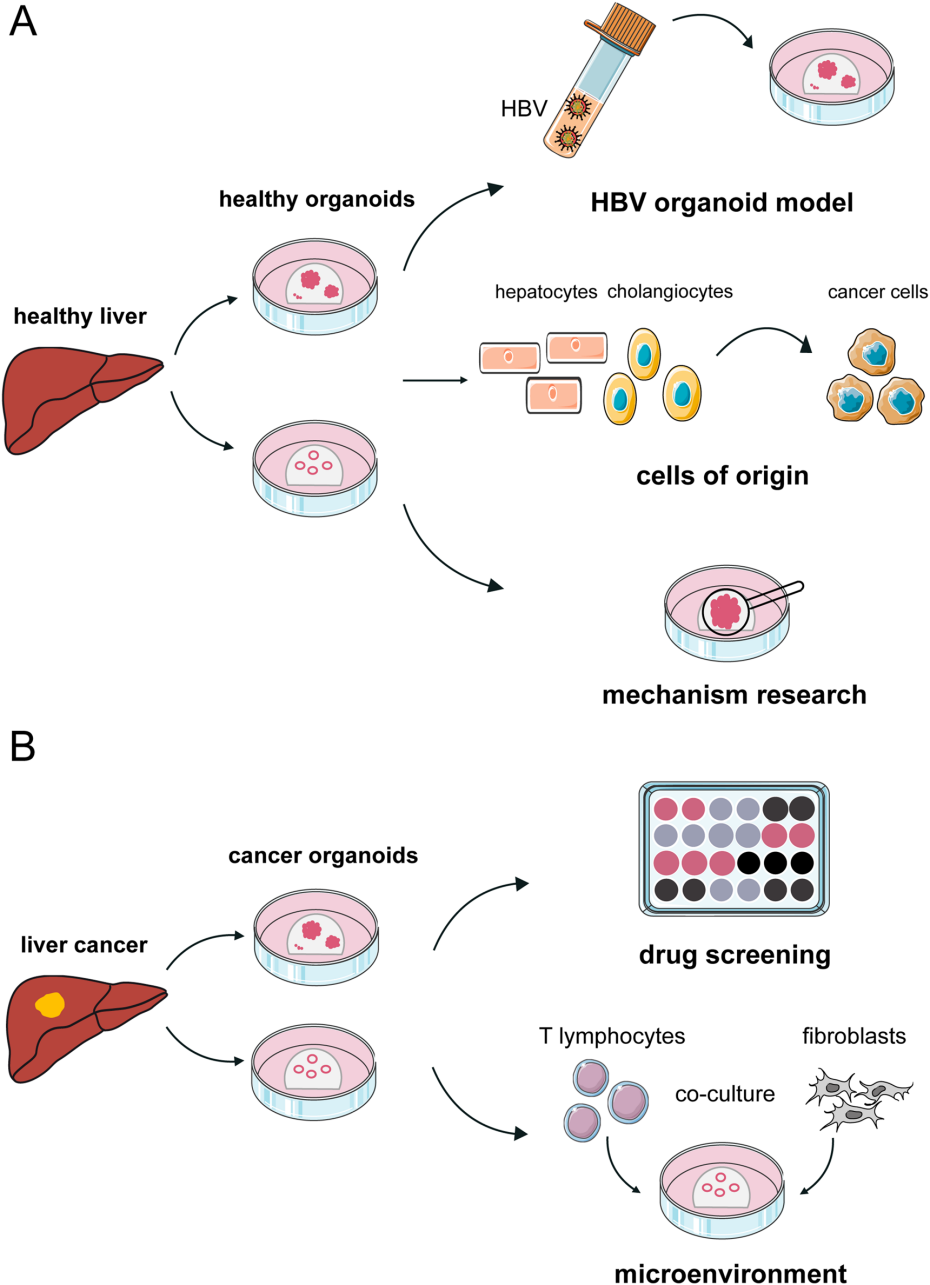
Applications of liver organoids. **A** Organoids derived from healthy liver tissues can be used as a model in basic research to investigate the cells of origin for liver cancer and the mechanism of carcinogenesis, especially in the HBV-associated liver cancers. **B** Organoids derived from liver cancer tissues can be expanded in vitro and cryopreserved enabling the establishment of biobanks. This is mainly used for drug screening in personalized medicine. Additionally, liver cancer organoids make it possible for the co-culture between cancer cells and other cells in microenvironment. Based on this technology, more basic research can be performed

Patient-derived organoids (PDOs), a particular kind of cancer organoids, is widely used for cancer research to mimic the situation of primary cancers, especially at the malignant stage. There are two main sources of PDOs, needle biopsies and human tumor specimens by surgery. The process of building PDOs is similar to that of organoids from the mouse PLC model, and the overall efficiency is 37.5% for tumor specimens by surgery and 26% for biopsy samples [19, 37]. Vlachogiannis et al. stated that no inverse correlation was observed between the PDO establishment rate and the presence of necrosis based on research of PDOs established from metastatic gastrointestinal cancers [38]. Besides, no correlation was found between PDO take-up rate and tumor percentage, suggesting that even in cases of a low tumor/stroma ratio, PDOs could also be established. This might aid in circumventing the shortage of next-generation sequencing (NGS) and is better suited for precision medicine, including drug screening and prediction of treatment responses (Fig. 3).

## Culture medium of liver organoids

### Basal medium

Advanced DMEM/F12 is a common basal medium used for mammalian cell culture in low concentrations of fetal bovine serum, which includes glucose, nonessential amino acids, sodium pyruvate, etc. When it is used for the culture of liver organoids, plenty of additional agents need to be supplemented (Table 2).

**Table 2:** Summary of culture medium compositions for liver organoids

	Function	Concentrations			
		Human		Mouse	
		Healthy	Cancer	Healthy	Cancer
Basal medium					
Advanced DMEM/F12	Providing basal nutrition				
HEPES	Maintaining the osmotic pressure	10 mM	10 mM	10 mM	10 mM
B27/N2	Suppressing cell differentiation, supporting the growth	1–2% B27 + 1% N2	2% B27 + 1% N2	2% B27	2% B27 + 1% N2
GlutaMAX	Common substitution of l-glutamine	1%	1%	1%	1%
*N*-Acetyl-l-cysteine	Regulate cell proliferation, differentiation and apoptosis	1–1.25 mM	1.25 mM	1.25 μM/1–1.25 mM	1.25 μM
Nicotinamide	Playing important role in self-renewal of HCC stem cells and cellular energy metabolism	10 mM	10 mM	10 mM	10 mM
Cytokines					
Wnt3a	Ligand of canonical Wnt/β-catenin pathway, supporting strongly increased self-renewal of organ and embryonic stem cells	800 ng/ml Wnt3a liposomes or 30% Wnt3a—condition medium	30% Wnt3a—condition medium	100 ng/ml or 30% Wnt3a—condition medium	30% Wnt3a—condition medium for initial 3 days
BMP7	Regulating cell proliferation, differentiation, migration, organization, and apoptosis	**25 ng/ml in DM**	**25 ng/ml in DM**		
EGF	Growth factor	50 ng/ml	50 ng/ml	50 ng/ml	50 ng/ml
FGF	Growth factor	100 ng/ml FGF10 ± 100 ng/ml FGF7 **(100 ng/ml FGF19 in DM)**	100 ng/ml FGF10 **(100 ng/ml FGF19 in DM)**	50–100 ng/ml FGF10 ± 50 ng/ml FGF7	100 ng/ml
HGF	Growth factor	25 ng/ml	25 ng/ml	25/40/50 ng/ml	50 ng/ml
Gastrin	Growth factor for colorectal, stomach, liver, and pancreatic cancer	10 nM	10 nM	10 nM	10 nM
Inhibitors					
Noggin	inhibitor of BMP-4 and BMP-7; to inhibit the differentiation of stem cells	**25 ng/ml in IM**	**25 ng/ml in IM**	100 ng/ml	10% Noggin-conditioned medium **for initial 3 days**
A83-01	ALK4/5/7 inhibitor; to decrease cell motility, adhesion and invasion increased by TGF-β1	2-5 μM **(0.5 µM in DM)**	5 μM **(0.5 µM in DM)**	1 μM **(50 nM in DM)**	
Y-27632	Rock inhibitor; to facilitate the attachment of primary cells in vitro, inhibit the apoptosis of embryonic stem cells and promote the self-renewal and proliferation of stem cells	10 μM	10 μM	10–20 μM	
CHIR99021	GSK3β inhibitor; to enhance Wnt target gene expression	3 μM		3 μM	
Activators					
R-spondin-1	Wnt pathway activator	1 μg/ml or 10–15% RSPO1—condition medium	10% RSPO1—condition medium	0.5–1 μg/ml or 15% RSPO1—condition medium	10% RSPO1—condition medium
Forskolin	Adenyl cyclase activator (cAMP activator)	10 μM	10 μM		
References		[24, 26, 49, 103]	[19, 37]	[16, 26, 103, 104]	[36]

Bold values represent the concentrations of additives in isolation medium or differentiation medium rather than expansion medium

*IM* isolation medium, *DM* differentiation medium

HEPES is a buffer commonly used in cell culture to maintain the osmotic pressure of the culture system. B27 is an optimized serum-free supplement usually used to support the growth of embryonic, post-natal, and adult hippocampal and other central nervous system neurons. For organoid culture, B27 may suppress cell differentiation. N2 is also a supplement for serum-free neurons with similar function to B27 [39].

Amino acids are the basic needs of cell culture. For organoid culture, three amino acids should be supplemented because of their short half-life: l-glutamine, *N*-acetyl-l-cysteine and nicotinamide. l-glutamine is one of the nonessential amino acids with unique metabolic functions. It is a precursor for the synthesis of other amino acids, proteins, nucleotides, and many other biologically important molecules. l-Glutamine also participates in cellular energy metabolism and intercellular adhesion [40–42]. While, its autodegradation in water limits the use of l-Glutamine in cell culture. GlutaMAX is the common substitution of l-glutamine, because of its stability in water. *N*-Acetyl-l-cysteine, a precursor of glutathione, is an effective antioxidant and free radical scavenger, and can activate the PI3K/Akt signaling pathway to regulate cell proliferation, differentiation and apoptosis [43]. Nicotinamide, a member of the B vitamin family, participates in cellular energy metabolism and plays an important role in the self-renewal of HCC stem cells [44]. Some studies have reported that nicotinamide could promote the expansion of organoids by inhibiting the activity of SIRT1 to suppress cell differentiation [45].

### Cytokines

Two different Wnt signaling cascades have been identified, called non-canonical and canonical pathways, the latter involving the β-catenin protein. The canonical pathway is an evolutionarily conserved signaling mechanism that regulates fundamental physiological and pathological processes. The role of the Wnt/β-catenin cascade in regulating liver homeostasis, regeneration, and tumorigenesis has been extensively reviewed [46–48]. Self-renewal of the stem cells in organoids requires activation of the Wnt pathway. Wnt3a is a key ligand of the canonical Wnt/β-catenin pathway and supports strongly increased self-renewal of organ and embryonic stem cells and the serum-free establishment of human organoids from healthy and diseased livers [49]. Similarly, R-spondin-1, an activator of the Wnt pathway, is also used for organoid culture to maintain high Wnt/β-catenin signaling activity [50]. It has been reported that R-spondin-1 is dispensable for the growth of liver organoids and costly, thus being removed from the culture medium [51].

Bone morphogenetic protein (BMP) belongs to the transforming growth factor beta (TGF-β) superfamily. BMP signaling cascades play a key role during embryonic development and maintenance of adult tissue homeostasis, as well as regulate cell proliferation, differentiation, migration, organization, and apoptosis [52, 53]. In the process of organoid development, BMP plays an active role [54, 55]. Noggin, an inhibitor of BMP-4 and BMP-7, suppresses the activation of BMP signaling cascades. Adding BMPs or removing Noggin, stem cells are lost from the cultures through differentiation towards adult cells [19, 56].

To maintain the growth and proliferation of organoids, certain growth factors are added to the culture medium. Epidermal growth factor (EGF) plays an irreplaceable role in signal transduction pathways that are involved in regulating cellular proliferation, differentiation, and survival by binding to its receptor, EGFR [57]. Fibroblast growth factors (FGFs) are a family of cell signaling proteins produced by macrophages. They participate in a wide variety of processes, especially as crucial elements for normal development in mammalian cells. A characteristic of FGFs is that they can bind to heparin and to heparin sulfate. Thus, some are sequestered in the extracellular matrix of tissues that contain heparin sulfate proteoglycans and are released locally upon injury or tissue remodeling [58]. Hepatocyte growth factor (HGF), secreted by mesenchymal cells, targets not only primarily epithelial cells and endothelial cells, but also haemopoietic progenitor cells and T cells. Its ability to stimulate mitogenesis, cell motility, and matrix invasion gives it a central role in angiogenesis, tumorigenesis, and tissue regeneration [59]. EGF, FGF and HGF are necessary growth factors for hepatocytes. It has been proven that hepatocytes can enter diffusely into proliferation under the influence of EGF, HGF and TGFα rather than other growth factors [60]. HGF can be used to promote liver stem cell expansion and rescue liver dysfunction combined with R-spondin-1 [61].

Gastrin and Forskolin are also widely used in liver organoid culture. Gastrin is an important peptide hormone in gastrointestinal tract, mainly secreted by G cells. It has been reported that gastrin is a growth factor for colorectal, stomach, liver, and pancreatic cancer [62]. Gastrin is customarily added to prolong the survival time of intestinal and liver organoids. Forskolin, an adenylate cyclase activator, is used to support liver organoids expansion [63, 64].

### Inhibitors

A83-01 is a potent inhibitor of TGF-β type I receptor ALK5 kinase, ALK4 and ALK7. It reduces the level of ALK-5-induced transcription and blocks the ALK4-TD- and ALK7-TD-induced transcription. A83-01 can decrease cell motility, adhesion and invasion increased by TGF-β1, but does not change cell proliferation [65].

A83-01 inhibits mesenchymal cells and supports epithelial cell growth whereas Y-27632 facilitates the attachment of primary cells in vitro [66, 67]. Y-27632 is an ATP-competitive inhibitor of ROCK-I and ROCK-II. Y-27632 can inhibit the apoptosis of embryonic stem cells by inhibiting ROCK and promote the self-renewal and proliferation of stem cells. To avoid the growth of noncancerous cells, R-spondin-1, Noggin and Wnt3A are removed by supplementation of the classical medium for liver organoid culture with dexamethasone and Y-27632 [68].

The GSK3β inhibitor CHIR99021 plays a regulatory role in a variety of signaling pathways such as TGF-β, Nodal and MAPK. Its effect on the Wnt/β-catenin pathway is dose-dependent. Low-dose CHIR is unable to support the survival of organoids. Increasing CHIR up to a high dose, enhanced Wnt target gene expression to a level similar to that of R-spondin1 treatment. Combining LDN-193189, a BMP type I receptor inhibitor, with CHIR could replace the role of Noggin in organoids [50].

## Liver organoids for carcinogenesis research

### Liver organoids to investigate HBV replication and HBV-associated carcinogenesis

**Fig. 4:**
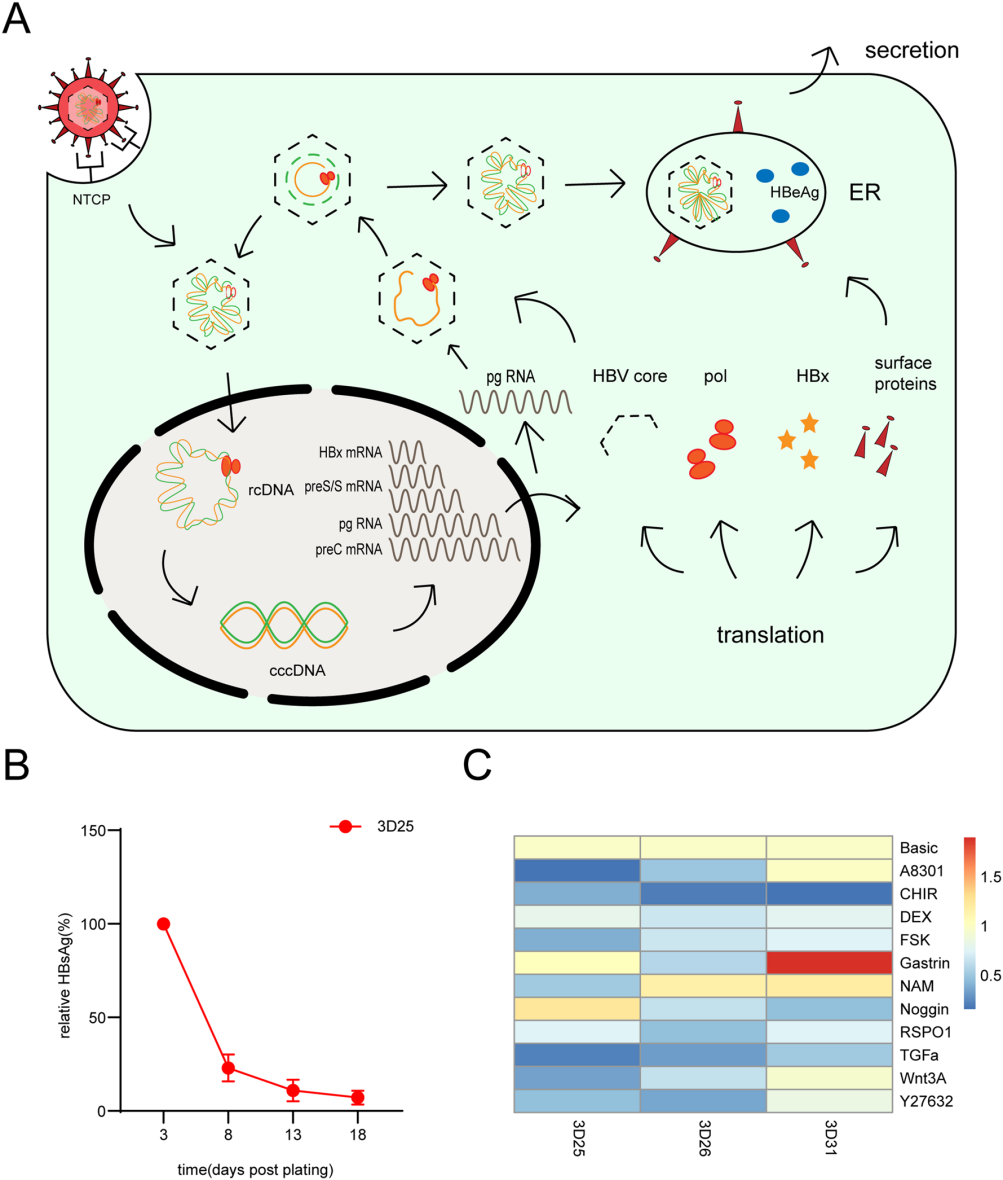
HBV life cycle and the unpublished data of suppression of HBV expression in the PDO model. **A** HBV life cycle. HBV enters hepatocytes by membrane fusion and internalization through NTCP, followed by uncoating, and nuclear transport of the RC DNA. The RC DNA is converted to cccDNA, which serves as a template for the transcription of pregenomic HBV RNA and generates 4 different mRNA transcripts: 3.5 kb preC RNA and pgRNA, 2.4 and 2.1 kb preS/S mRNAs, and 0.7 kb HBx mRNA. These RNAs are exported to the cytoplasm, and plenty of HBV proteins are generated. pgRNA is selectively packaged inside core particles, followed by several progresses to generate RC DNA. Following viral assembly in the endoplasmic reticulum, mature HBV is released into the extracellular matrix. **B** Time course of relative HBsAg levels in expansion medium in the PDO model generated from patients infected with HBV. Error bars represent the mean ± SEM. **C** Heatmap of NTCP mRNA levels relative to GAPDH of different supplements added into basic medium with HEPES, B27, N2, Glutamax, *N*-acetyl-l-cysteine, EGF, FGF10 and HGF

It is estimated that 5% of the world population, or approximately 400 million people, are currently chronically infected with HBV [69, 70]. Moreover, HBV infection is the most prominent risk factor for HCC development, accounting for approximately 50% of cases globally in 2015 [71]. HBV is a member of the hepadnaviridae family, with an approximately 3.2 kilobase (kb) pair of partially double-stranded circular DNA genome. And this genome is attached to the viral polymerase at the 5′ end of the (-) strand [72]. When HBV infects hepatocytes, the pre-S domain of HBsAg is attached to sodium taurocholate co-transporting polypeptide (NTCP). Then, the virus enters cells by membrane fusion and internalization [73, 74] (Fig. 4A). Upon infection of hepatocytes, relaxed circular DNA (RC DNA) is released and converted to the covalently closed circular (ccc) DNA form, although the underlying mechanism is unknown. The cccDNA, which forms a minichromosome with high stability, originates at the initiation of the viral life cycle [75]. This minichromosome serves as a template for the transcription of pregenomic HBV RNA and generates 4 different mRNA transcripts (3.5 kb, 2.4 kb, 2.1 kb and 0.7 kb) [76]. Based on these findings, plenty of HBV proteins are generated, followed by viral assembly in the endoplasmic reticulum. After nucleocapsid and HBV surface proteins are packaged, mature HBV is released into the extracellular matrix.

Currently, models of HBV research can be divided into three kinds: cellular models, animal models and organoid models. In general, cellular models are unsuitable for HBV-associated carcinogenesis research, although they easily maintain HBV replication and cost little. Animal models, especially primates, support HBV infection and immune responses but are so expensive and too difficult to maintain. Compared with the two models mentioned above, organoid models are amenable to genetic modification and cost moderately. Moreover, organoids support the full HBV life cycle and are suitable for research on HBV-associated carcinogenesis [77]. To date, two organoid models have been established that describe the potential of liver organoids to study HBV infection and replication in vitro; the pluripotent stem cell-derived model by Nie et al. [78] and the adult stem cell-derived model by Crignis et al. [79] Nie et al. established a functional liver organoid (LO) model from human induced pluripotent stem cells (hiPSCs). In a 3D culture system, hiPSC-derived endodermal, mesenchymal, and endothelial cells are co-cultured with a certain medium. Based on cell–cell interactions, self-organization appears and functional organoids are generated when the cells mentioned above differentiate. These functional hiPSC-LOs can support the full HBV life cycle and mimic HBV-associated hepatic dysfunction, truly recapitulating host-virus interactions. Additionally, Crignis et al. proved that a liver organoid model may support HBV replication in vitro. Liver organoids are generated from healthy donors. Then, these organoids were efficiently infected with both recombinant virus and HBV-infected patient serum. Excitingly, HBsAg, HBeAg, HBV core proteins, and even cccDNA can be detected in the culture supernatant of organoids. In addition, they also generate polyclonal liver organoids transducted by lentivirus. This model can efficiently produce HBV in a certain expansion medium.

HBV contributes to HCC development through direct and indirect mechanisms. Reported mechanisms of HBV infection leading to the development of hepatocellular carcinoma mainly include: genomic instability caused by partial integration of viral DNA into the host cell genome, insertional mutations of cancer-related genes, and promoting transformation of truncated HBx and preS/S proteins. In addition, genome damage, abnormal regulation of the cell cycle, apoptosis and other signaling pathways caused by HBV long-term massive replication and massive expression of HBx and other viral proteins, as well as liver damage and liver fibrosis caused by the imbalance of the liver immune microenvironment are also important for the occurrence and development of HBV-associated liver cancers [80]. HBV genome integration affects the abnormal expression of a series of cancer-related genes, such as proliferation, apoptosis, transcription, development and differentiation through cis-acting [81–83]. Moreover, viral genome integration into virus-host chimeric proteins promotes cell cycle progression and hepatocellular carcinogenesis [84].

Because of the ability of liver organoids to support the full HBV life cycle, it is possible to study the roles of these viral proteins during the natural course of infection. And it comes true that investigating the downstream target genes and carcinogenesis mechanisms in a primary hepatocyte system by the exogenous expression of HBx protein or other candidate viral/host proteins in hepatocytes, because organoids can be genetically modified. Interestingly, Crignis et al. generated HBV-infected PDOs from non-tumor cirrhotic tissue of explants from liver transplant patients. Transcriptomic analysis of those organoids indicates that an aberrant early cancer gene signature exists, which clustered with the HCC cohort on The Cancer Genome Atlas Liver Hepatocellular Carcinoma dataset and away from healthy liver tissue. This clarifies the potential that HBV-infected PDOs may provide evidence of some invaluable novel biomarkers for HBV-associated carcinogenesis and cancer development in patients infected with HBV [79].

**Fig. 5:**
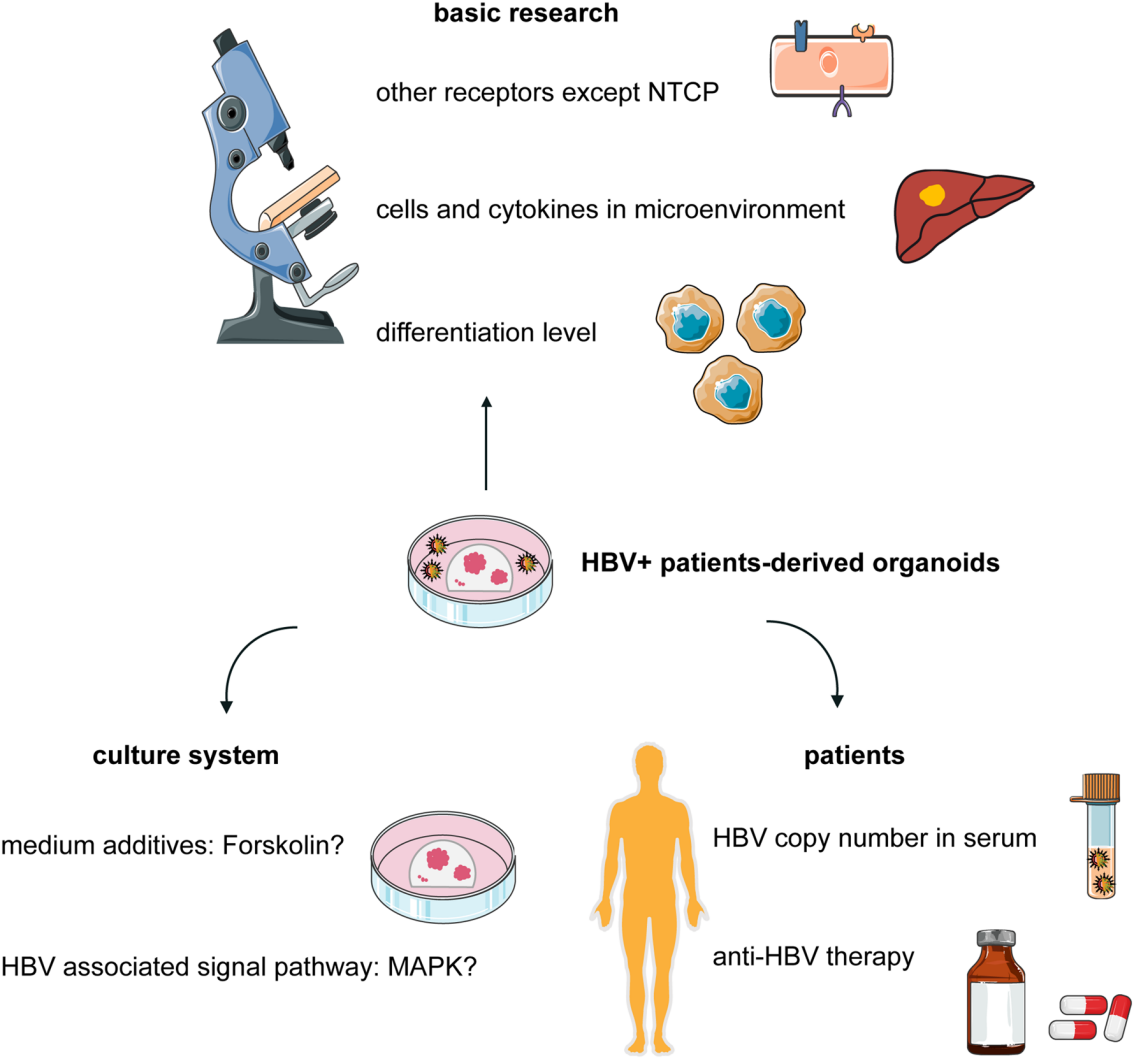
Unknown features of patient-derived organoids from HBV+ patients. In basic research of HBV, whether there are other receptors except NTCP is still unknown. The differentiation level of HBV host cells also may influence the entering process of HBV. The role that other cells and cytokines in microenvironment play in the HBV life cycle requires more research. And whether the current culture system of PDOs is suitable for HBV replication or not remains unclear. More research is required to clarified the influence of additives in culture medium on HBV replication and the activity of HBV-associated signaling pathway. Before the culture of organoids, patients usually get anti-HBV therapies. The influence of these therapies remains unknown. The correlation between HBV copy number in serum and in culture medium requires more studies to clarify

Organoids used for HBV research are only healthy ones rather than cancers. We can successfully study HBV replication or HBV-associated carcinogenesis by infecting healthy organoids exogenously. In fact, it is still a great challenge to establish a patient-derived organoid model from those infected with HBV that can stably maintain HBV replication in vitro. This type of organoid model may not only maintain the biological characteristics of the original cancer but also show the infection and replication of HBV endogenously on a cancer background. In our trial, in expansion medium, the expression of HBV was suppressed continually in the PDO model generated from patients infected with HBV (Fig. 4B). Crignis et al. reported that differentiated organoids maintained in differentiation medium are more efficiently infected and produce higher viral titers than those in expansion medium [79]. This indicates that organoids in differentiation medium seem to support HBV replication and expression. Furthermore, whether HBV infection and expression are affected by culture medium composition is unknown. We have proven that the expression of NTCP is unstable and unrepeatable between different organoids by changing the culture medium composition (Fig. 4C). However, it has been reported that the presence of NTCP alone on the cell membrane may not automatically make cells more conducive to infection [85]. These results imply that viral internalization may require some highly probable but currently unknown host factors. The role these unknown factors play in the suppression of HBV expression in organoid culture is difficult to clarify (Fig. 5). We assume that the culture system for liver PDOs may not support HBV replication and expression. This means that some compositions block the signaling pathway necessary for the HBV life cycle or that some compositions are lacking support. The expression of HBV can be maintained for a long time, although primary human hepatocytes (PHHs) are infected with HBV in the medium with Forskolin [86]. However, Forskolin may repress the expression of NTCP to suppress HBV infection in PDO culture (Fig. 4C). Whether this contradiction is led by other supplements, such as SB431542, IWP2, DAPT, and LDN193189, in PHH culture remains unknown. It has been demonstrated that the MAPK signaling pathway is closely related to HBV replication and expression. P38 MAPK is activated by HBsAg, and HBsAg production is attenuated by inhibiting the pathway [87]. HBx activates the p38 MAPK pathway, which in turn facilitates HBx-mediated STAT3 phosphorylation [88], while STAT3 directly binds to HBV X/EnhI to promote HBV replication [89]. HoxA10 interacts with p38 MAPK and recruits SHP-1 to repress HBV replication, as well as HoxA10 binds to the EnhI/X promoter and competes with STAT3 to attenuate HBV transcription [90]. Under culture of liver PDOs, whether the MAPK signaling pathway is repressed remains unclear. The study for establishing the model of liver PDOs with endogenous HBV expression may be a new starting point for HBV infection. Based on this model, more research can be performed to clarify the role that HBV plays in liver oncobiology.

### Liver organoids for research on carcinogenesis without hepatitis virus

Although several studies have reported the cell origin and extracellular matrix of liver cancer, carcinogenesis is still unclear. With the explorations of liver organoids, our understanding of liver carcinogenesis may witness more advancement. Naruse et al. established an organoid-based carcinogenesis model induced by in vitro chemical treatment [34]. In their study, the model established with mouse tissue-derived organoids and diethylnitrosamine (DEN)-treated organoids showed carcinogenic histopathological characteristics, which could be applied to investigate early molecular events to clarify the carcinogenesis driven by chemical factors.

Moreover, Sun et al. described the first documentation of human hepatocytes into iCCA cells [31]. Organoids with liver architecture and function were established using directly reprogrammed human hepatocytes. Then, they observed excessive contact between mitochondria and endoplasmic reticulum membranes with the introduction of c-MYC which might be an unrecognized oncogenic event in liver carcinogenesis [91]. By tracing the process of carcinogenesis induced by RAS in hepatocyte organoids, hepatocytes expressed several characteristics that are unique in iCCA cells. Similarly, some studies stated that mutations affecting BAP1 might be involved in the carcinogenesis of iCCA [35, 92].

To interrogate the mechanism of biliary carcinogenesis, another model was established using organoids from murine liver and gallbladder [93]. The organoids were transduced with lentiviral vectors to reconstitute genetic alterations common in biliary tract cancers, and then inoculated into immunodeficient mice. Their results showed that mutant Kras could drive the development of iCCA and this model might accelerate research on carcinogenesis.

## Drug screening and personalized medicine

As HCC is a cancer rich in heterogeneity among patients, and even within cancer cells derived from the same individual, personalized treatment is of great importance to meet the tenets of precision medicine. Some studies have proven that tumor organoids derived from human PLC retain features from their tissue of origin [19, 37], indicating it holds promise to leverage organoids, especially PDOs, in personalized medicine.

By establishing PDOs, it was demonstrated that miRNA21 appeared to mediate the resistance of CCA cells to HSP90 inhibitors and that HSP90 inhibitors might be developed for the treatment of CCA [94]. Hedgehog signaling and receptor tyrosine kinase-induced reactivation of the MEK/ERK and AKT signaling pathways might be related to sorafenib resistance [95, 96]. These studies show a perspective for research on drug resistance and personalized medicine.

The potential of tumor organoids in drug screening for HCC was stated by Li et al. [97]. They established multiple cancer organoid lines from distinct regions of the tumor for each primary human liver cancer surgical specimen. Then, 129 drugs were used for the treatment of 27 lines. As a result, a subset of drugs appeared pan-effective, displaying at least moderate activity in the majority of these cancer organoid lines, although the majority of drugs were either ineffective, or effective only in selected lines.

Some efforts have been made in the clinical use of PDOs from metastatic colorectal and gastroesophageal cancer, suggesting that PDOs can recapitulate patient responses and be implemented in personalized medicine programs [38]. It is worth anticipating the widespread use of organoids from PLCs in personalized medicine.

## Conclusions and futures

Although many studies have concentrated on the cell origin and carcinogenesis of liver cancer by means of organoids, cancer microenvironment cells, such as immune cells and vascular cells, have been insufficiently explored. Efforts have been made in the co-culture of human induced pluripotent cells with human adipose microvascular endothelial cells [98], rat hepatocytes with rat stellate cells [99], especially human pancreatic cancer organoids with stromal and immune cells [100], and tumor organoids with peripheral blood lymphocytes [101]. More research could be conducted on the microenvironment under hepatitis B infection and antitumor immunity using liver organoids.

Liver organoids are widely used for investigations of various liver diseases, including hepatitis, chronic liver diseases, metabolic diseases, and liver cancer. However, most studies focus on adult liver disorders rather than infant illness. In the future, more attention could be paid to the generation of fetal hepatocyte organoids and hepatoblastoma organoids, the most common cancer of fetal livers [91].

In addition, nearly all studies of liver cancer using organoids are about PLC. However, metastatic hepatic carcinoma and metastatic tumors of the liver are rarely studied. Skardal et al. inoculated liver organoids with colon carcinoma cells to create liver-tumor organoids for in vitro modeling of liver metastasis [102]. This 3D liver-tumor organoid was proven to be a better model of metastatic tumors than 2D cell cultures. Although this kind of model could not mimic the actual situation of metastatic tumors of the liver completely, it is worth further research.

In this review, we summarize the categories and development of liver organoids, especially focus on the functions of culture medium components and applications of organoids for HBV infection and HBV-associated liver cancer studies. Moreover, we provide insights into a potential patient-derived organoid model from those infected with HBV based on our study. In summary, although the size of organoids and the time of culture impede the applications of organoids in liver cancer research, there is still substantial potential for research on organoids in liver cancer. Developing a biobank of liver cancer and precision medicine also requires more trials.

## Data Availability

All data used in this study are public.

## Abbreviations

cccDNA: Covalently closed circular DNA
DEN: Diethylnitrosamine
EGF: Epidermal growth factor
FGF: Fibroblast growth factor
GEMM: Genetically engineered mouse model
HBV: Hepatitis B Virus
HCC: Hepatocellular carcinoma
HGF: Hepatocyte growth factor
iCCA: Intrahepatic cholangiocarcinoma
iPSC: Induced pluripotent stem cell
Lgr5: Leucine-rich repeat-containing G-protein-coupled receptor 5
LO: Liver organoid
NGS: Next-generation sequencing
NTCP: Sodium taurocholate co-transporting polypeptide
PDO: Patient-derived organoid
PDX: Patient-derived xenograft
PLC: Primary liver cancer
RC DNA: Relaxed circular DNA
TGF-β: Transforming growth factor beta
